# High variation in the response of calves to a low-dose lipopolysaccharide challenge is associated with early-life measurements

**DOI:** 10.3168/jdsc.2023-0437

**Published:** 2024-01-15

**Authors:** M.S. Gilbert, A. Lammers, W.J.J. Gerrits

**Affiliations:** 1 Animal Nutrition Group, Wageningen University & Research, 6700 AH, Wageningen, the Netherlands; 2 Adaptation Physiology Group, Wageningen University & Research, 6700 AH, Wageningen, the Netherlands

## Abstract

Lipopolysaccharide (LPS) challenges are commonly used in animal studies as a model for infection with gram-negative bacteria and innate immune activation. We used a low-dose LPS challenge for evaluating interindividual variation in innate immune responses in calves. This was part of a larger study aimed at predicting interindividual variation in feed efficiency in veal calves by variation in feeding motivation, digestion, metabolism, immunology, and behavioral traits. However, due to unexpected high mortality, this LPS challenge was performed in 32 calves rather than in 130 calves, which was initially intended in that larger study, and the 32 calves subjected to the LPS challenge were removed from that larger study. The objective of this short communication is to report the effects of a low-dose LPS challenge in those 32 calves and to examine whether the high variation in calves' responses to LPS could be explained by parameters related to feeding motivation, digestion, behavior, and immunology measured in early life. Thirty-two male Holstein-Friesian calves of Dutch origin were intravenously injected with LPS (0.05 μg/kg of body weight) at an age of 72 ± 0.6 d. Rectal temperature and respiratory frequency were recorded before injection and every hour after injection up to 6 h. In the 8 wk before the LPS challenge, measurements were performed related to general health, feeding motivation, digestion, behavior, and immunity. Following LPS administration, 3 calves died of shock, a fourth calf was euthanized because of severe symptoms of shock and 3 other calves were treated with corticosteroids to counteract observed symptoms of shock. Within the group of 25 relatively mild-responding calves, large interindividual variation in clinical responses to LPS was observed. The maximum increase in rectal temperature varied from 0.6 to 1.9°C and averaged 1.2 ± 0.39°C (coefficient of variation was 32%). The maximum increase in respiratory frequency varied from 16 to 132 bouts/min and averaged 60 ± 28 bouts/min (coefficient of variation was 48%). Little differences were found in early-life measurements between the 7 heavy and 25 mild responders, although heavy responders tended to have a better umbilical hernia score, and had a lower score in a human approach test (i.e., were less reactive) and lower presence of fecal pathogens. The maximum increase in rectal temperature correlated negatively with blood hemoglobin concentration at arrival of the calves at the facilities (r = −0.59) and in wk 4 (r = −0.53). The maximum increase in respiratory frequency correlated negatively with fecal color score (r = −0.43) and positively with fur score in wk 5 (r = 0.50). Overall, mortality (12.5%) and variation in clinical response was high after a low-dose LPS challenge in clinically healthy calves and some hematological and health measurements in early life were related to the clinical response of calves to LPS.

## Graphical Abstract

**Figure:**
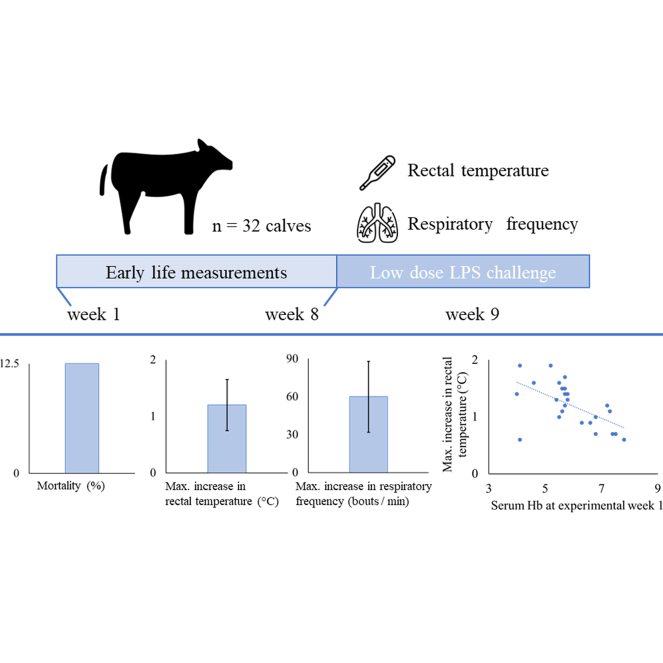

Lipopolysaccharide or endotoxin is part of the outer membrane of gram-negative bacteria, such as *Escherichia coli*. Clinical effects of intravenous exposure to LPS include diarrhea, increased respiratory frequency and heart rate, fever, and endotoxic shock (Jacobsen et al., 2005; Borderas et al., 2008). Lipopolysaccharide challenges are commonly used in animal studies as a model for infection with gram-negative bacteria and innate immune activation. These challenges are used, for instance, to study behavioral changes during illness (Borderas et al., 2008) or to determine effects of diet on immune responses (Burdick et al., 2012).

The response of animals to LPS might depend on LPS serotype and dose (Jacobsen et al., 2005; Migale et al., 2015). Calves are sensitive to LPS, with intraperitoneal administration of 0.025 mg/kg of BW being lethal for calves, compared with 5 and >50 mg/kg of BW for pigs and poultry, respectively (Berczi et al., 1966). Therefore, lower dosages of LPS are typically used in calves (i.v., 0.025–0.05 µg/kg of BW, Borderas et al., 2008) compared with those used in dairy cows (i.v., 1.0 μg/kg of BW, Oh et al., 2017), pigs (i.v., 25 μg/kg of BW, Roh et al., 2015), and broilers (i.p., 500 μg/kg of BW, Zheng et al., 2020). These low dosages of LPS (i.e., 0.025 and 0.05 µg/kg of BW) elicited responses in rectal temperature, respiratory frequency, and sickness behavior in dairy calves (Borderas et al., 2008). Therefore, we performed a LPS challenge using the same dose (i.v. 0.05 µg/kg of BW) and serotype for evaluating interindividual variation in innate immune responses in calves. Initially, this challenge was included as a measurement in a larger study aimed at predicting interindividual variation in feed efficiency in veal calves by variation in feeding motivation, digestion, metabolism, immunology, and behavioral traits (Gilbert et al., 2017), which was approved by the Animal Care and Use Committee of Wageningen University (Wageningen, the Netherlands; registration number 2014041.e). However, due to unexpected high mortality, this LPS challenge was stopped immediately. Therefore, the LPS challenge was performed for a smaller number of calves than initially intended in that larger study and the calves subjected to the LPS challenge were removed from that experiment. These events have been thoroughly discussed with the animal welfare officer and reported to the Animal Care and Use Committee of Wageningen University. The objective of this short communication is to report the effects of a low-dose LPS challenge in calves and to examine whether the high variation in calves' responses to LPS could be explained by parameters related to feeding motivation, digestion, behavior, and immunology measured in early life.

Thirty-two male Holstein-Friesian calves of Dutch origin sourced from a collection center were housed individually on wooden, slatted floors (1.2 m^2^ per calf). At arrival at the research facilities, calves were 16 ± 3.4 d of age and weighed 44 ± 3.0 kg (mean ± SD). Calves were fed milk replacer and solid feed (concentrates, rapeseed straw, and alfalfa in a 70:15:15 ratio) according to a practical veal calf feeding scheme (for details, see Gilbert et al., 2017). At an age of 72 ± 3.4 d (mean ± SD), calves were injected with LPS (from *E. coli* O55:B5, L6529, Sigma-Aldrich, St. Louis, MO; dissolved in sterile saline solution at a concentration of 1 μg/mL) in the jugular vein at 0.05 μg/kg of BW. All calves had a prechallenge rectal temperature of <39.3°C. Rectal temperature and respiratory frequency were recorded before injection and every hour after injection up to 6 h and rectal temperature was again recorded at 8 h postinjection. All calves received the LPS on the same day between 0900 and 1000 h in the morning. The temperature and humidity averaged 26 ± 2.6°C and 71 ± 8.7% (mean ± SD), respectively, on the day of the LPS challenge.

In the 8-wk period before the LPS challenge, measurements related to feeding motivation, digestion and behavior were performed to characterize individual calves. In addition, hematological parameters and natural antibody titers (IgG and IgM binding keyhole limpet hemocyanin) were measured in the blood samples collected upon arrival. These early-life measurements and analyses have been described in detail (Gilbert et al., 2017). General measurements included BW (4-wk interval), blood hemoglobin (**Hb**) concentration (4-wk interval), rectal temperature (2-wk interval), and a veterinary health check (in wk 5). Medical treatment was applied when required based on clinical signs of illness and all treatments were recorded in this 8-wk period. The temperature and humidity averaged 21 ± 3.6°C and 71 ± 11.4% (mean ± SD), respectively, during this 8-wk period.

Three calves died of shock following the low-dose LPS challenge (2 calves at <1 h postinjection, 1 calf at <2 h postinjection). The second calf that died had received corticosteroids (i.m.; 0.6 mg/kg of BW of Rapidexon 2 mg/mL) and the third calf had received epinephrine (i.m.; 2 mg of adrenaline 1 mg/mL) to try to counteract shock. A fourth calf, that had already received epinephrine, was euthanized because of severe symptoms of shock (at 2.5 h postinjection). Three other calves were treated with corticosteroids to counteract observed symptoms of shock and prevent death. These 7 calves were grouped as heavy responders. The remaining calves (n = 25) were grouped as mild responders. For the mild responders, the maximum increases in rectal temperature and respiratory frequency following LPS administration were calculated for each calf from the prechallenge and maximum values.

Measurements related to feeding motivation, digestion, behavior, immunity, and general health (see Table 1 for descriptive data) conducted in the 8-wk period before the LPS challenge were used to test whether these early-life measurements differed between responder types using the TTEST procedure of SAS 9.4 (SAS Institute Inc., Cary, NC), with individual calf as experimental unit. If variances were unequal, the Cochran approximation option was used. Furthermore, to evaluate whether early-life measurements related to responses to LPS within the mild responder calves, the maximum increase in rectal temperature and the maximum increase in respiratory frequency were correlated with the early-life measurements using the CORR procedure with Spearman option. Results are presented as means ± standard deviation, and differences were considered significant when *P* < 0.05 and considered a trend when 0.05 ≤ *P* < 0.10. No calves or data points were excluded from the statistical analyses.

**Table 1:** Descriptive data of measurements performed in early life of calves (n = 32)^1^

Item	Mean	SD	CV
Hematological parameter upon arrival of calves at facilities			
Hematocrit (%)	31.9	6.80	21.3
Hemoglobin (mmol/L)	5.9	1.20	20.3
IgG titer^2^	3.3	1.67	49.5
IgM titer^2^	6.7	1.10	16.4
Feeding motivation			
Milk replacer intake^3^ (kg)	10.3	1.37	13.2
Drinking speed (kg of milk replacer/min)	4.3	2.00	19.6
Digestion			
Fecal pH	7.3	0.43	5.9
Fecal consistency score^4^	3.0	0.40	13.4
Fecal color score^4^	3.7	0.27	7.4
Total-tract retention time^5^ (h)	12.6	2.47	19.6
Behavior			
Human approach test^6^			
Approach phase, score (0 or 1)	0.1		
Touch phase, score (1 to 5)	2.9		
Activity^7^ (% of time standing)	13.7	5.32	38.9
Other			
Calf shape^8^ (cm^2^/kg of BW)	137.0	4.98	3.6
Fecal pathogens^9^ (0 to 4)	0.2		
Hemoglobin (mmol/L; wk 4)	5.3	0.88	16.6
Veterinary health check^10^			
Lung score	4.2	0.86	20.5
Umbilical score	3.9	0.64	16.4
Condition score	3.1	0.55	17.7
Fur score	4.1	0.55	13.4

1 All measurements and analytical procedures were described in detail in Gilbert et al. (2017).

2 Natural antibody titers, binding keyhole limpet hemocyanin (KLH), were measured.

3 Reconstituted milk replacer intake (at a concentration of 125 g/kg) during a single ad libitum intake test in which voluntary milk replacer intake was measured during 10 min.

4 Feces was scored 8 times during a 8-wk period on consistency (1–5: thin, normal/thin, normal, firm/normal, and firm) and color (1–7: white, yellow, light brown, brown, dark brown, gray, and black).

5 Total-tract retention time was determined by adding an indigestible colored marker (CrCl_3_ hexahydrate) to the milk replacer meal and scoring the first appearance of the green color in the feces by hourly scan sampling for 28 h.

6 A human approach test (Lensink et al., 2003) consisting of 2 phases was performed twice and the 2 scores were averaged per phase. In the approach phase, it was noted whether a drinking calf stopped drinking when a person approached. In the touch phase, the withdrawal reaction of the calf to the outstretching arm of the approaching person was scored from 1 (no withdrawal) to 4 (strong withdrawal reaction). For the approach phase, the number calves with score 0, 0.5, and 1 were 27, 4, and 1, respectively. For the touch phase, 3 calves scored 1, 2 calves scored 1.5, 2 calves scored 2, 7 calves scored 2.5, 5 calves scored 3, 4 calves scored 3.5, and 9 calves scored 4.

7 Activity of calves was measured during 3 d using scan sampling, in which the posture (standing or lying) of the calf was scored 2.5 h after the morning feeding and 2.5 h before the afternoon feeding. Activity was expressed as the percentage of scans in which the calf was standing of the total 48 scans per calf.

8 Calf shape was assessed twice, based on the measured heart girth and body length to calculate the surface area of the body [assuming a cylinder and calculating surface area as (2π × r2) + (π × d × h), where r = heart girth/2π, d = 2 × r, and h = body length], divided by BW.

9 One rectal fecal sample collected in wk 5 was analyzed for the presence of pathogens (rotavirus, coronavirus, *Escherichia coli*K99, and *Cryptosporidium*) using an immunoassay (BIO K 288, Bio-X Diagnostics, Rochefort, Belgium). The number of calves with no pathogens detected (score 0) was 27, and the number of calves with 1 pathogen detected was 5. There were no calves with 2, 3, or 4 pathogens present in the feces.

10 In wk 5, all calves were assessed by a veterinarian. Scores (1–5) were provided for the condition of the lungs (using a stethoscope with score 1: abnormal bronchial breathing (e.g., crackles, wheezes), increased respiratory frequency; and score 5: no abnormal breathing, no abnormal lung sounds, normal respiratory frequency), umbilicus (with score 1: umbilical hernia, inflammation; and score 5: completely closed umbilicus, no signs of inflammation), general condition (score 1: emaciated; and score 5: muscular), and fur (score 1: dull, curling fur; and score 5: smooth, shiny fur).

Average daily gain in the first 4 wk after arrival did not differ between responder types (*P* = 0.86) and averaged 480 ± 66 g/d. Heavy responders tended to have a higher (healthier) umbilical hernia score compared with mild responders (4.3 ± 0.5 vs. 3.8 ± 0.6, respectively; *P* = 0.076), as assessed by a veterinarian in wk 5. Mild responders had a higher score in phase 1 (approach phase) in the human approach test (0.12 ± 0.26; *P* = 0.031), where score 1 means a calf stops drinking milk replacer upon human approach. Furthermore, mild responders had a higher fecal pathogen presence (0.2 ± 0.4; *P* = 0.022) compared with heavy responders, mainly caused by zero values in the heavy responder group. The average rectal temperature (38.7 ± 0.19°C; *P* = 0.13) and individual antimicrobial supply against respiratory disease (*P* = 0.88) during the 8-wk period did not differ between responder types.

The responses in rectal temperature and respiratory frequency are presented in Figure 1 and correlations with early-life measurements are presented in Table 2. The maximum increase in rectal temperature in the mild responders varied from 0.6 to 1.9°C and averaged 1.2 ± 0.39°C (CV was 32%) and correlated negatively with blood Hb concentration (r = −0.59, *P* = 0.002) and hematocrit percentage at arrival (r = −0.51, *P* = 0.009), Hb concentration in wk 4 (r = −0.53, *P* = 0.007), and tended to correlate negatively with the prechallenge rectal temperature (r = −0.40, *P* = 0.050). The maximum increase in respiratory frequency in the mild responders varied from 16 to 132 bouts/min and averaged 60 ± 28 bouts/min (CV was 48%) and correlated negatively with fecal color score (r = −0.43, *P* = 0.031) and positively with fur score as assessed by a veterinarian in wk 5 (r = 0.50, *P* = 0.012). Respiratory frequency at 6 h after LPS injection was still above prechallenge value (Tukey adjusted *P* = 0.006).

**Figure 1:**
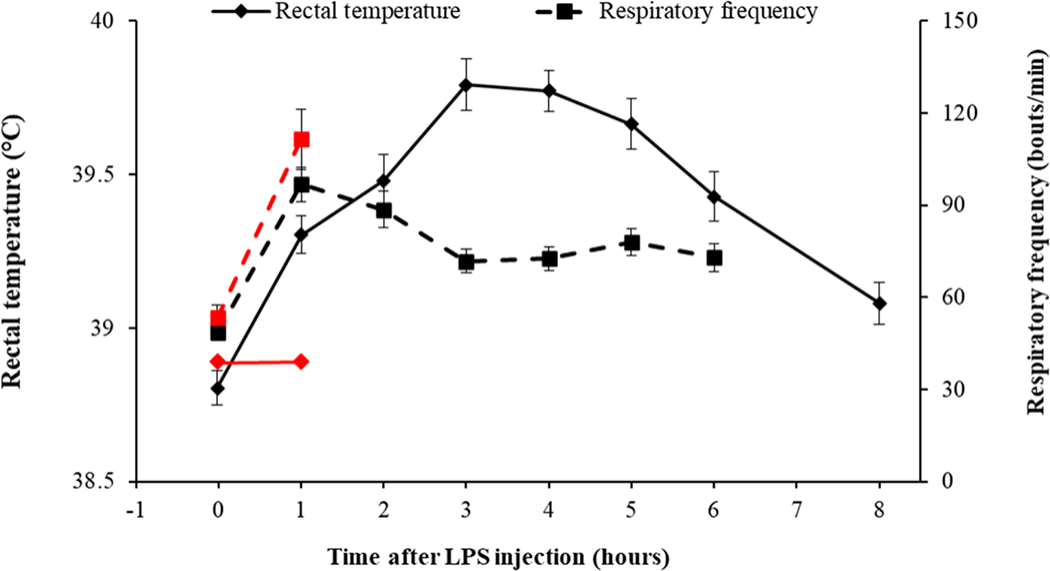
Rectal temperature and respiratory frequency response in calves subjected to an intravenous lipopolysaccharide challenge. Red markers represent calves that died of shock, were euthanized, or received corticosteroids to counteract observed symptoms of shock (n = 7 for time = 0 and n = 4 remaining calves at time = 1 h). Black markers represent calves (n = 25) that did not die or receive corticosteroids. Error bars represent SE.

**Table 2:** Spearman correlation coefficients (r) between early-life measurements and the maximum response in rectal temperature and respiratory frequency of calves (n = 25) subjected to an intravenous lipopolysaccharide challenge

Item	Maximum response in rectal temperature		Maximum response in respiratory frequency	
	r	*P*-value	r	*P*-value
Hematological parameter upon arrival of calves at facilities				
Hematocrit (%)	−0.51	0.009	−0.03	0.88
Hemoglobin (mmol/L)	−0.59	0.002	0.16	0.44
IgG titer	0.00	1.00	0.22	0.29
IgM titer	0.06	0.76	−0.23	0.26
Feeding motivation				
Milk replacer intake (kg)	−0.24	0.25	0.15	0.47
Drinking speed (kg milk replacer/min)	−0.23	0.26	0.29	0.16
Digestion				
Fecal pH	0.02	0.92	−0.17	0.41
Fecal consistency score	0.09	0.66	−0.12	0.58
Fecal color score	0.05	0.79	−0.43	0.031
Total-tract retention time (h)	0.13	0.53	−0.16	0.45
Behavior				
Human approach test				
Approach phase, score (0 or 1)	0.17	0.41	−0.26	0.21
Touch phase, score (1 to 5)	0.05	0.81	−0.21	0.31
Activity (% of time standing)	−0.15	0.48	0.12	0.56
Other				
Calf shape (cm^2^/kg of BW)	−0.08	0.70	0.03	0.91
Fecal pathogens (0 to 4)	0.20	0.33	0.15	0.47
Hemoglobin (mmol/L; wk 4)	−0.53	0.007	0.02	0.94
Veterinary health check				
Lung score	−0.12	0.57	−0.09	0.68
Umbilical score	−0.20	0.34	−0.05	0.81
Condition score	−0.09	0.68	0.20	0.34
Fur score	−0.30	0.14	0.50	0.012

In this study, mortality rate was unexpectedly high (12.5%) after a low-dose LPS challenge in calves. In contrast, none of the 15 calves died after an i.v. LPS (O55:B5) challenge at 0.025 or 0.05 µg/kg of BW in the study of Borderas et al. (2008), using LPS of the same *E. coli* serotype and dose as in the current study. Furthermore, no pre-shock states were observed in their pilot study (number of calves not specified; Borderas et al., 2008). One out of 24 Brahman bulls died after an i.v. LPS (O111:B4) challenge at 0.5 µg/kg of BW and in one bull the challenge was intervened (treatment is not specified) to prevent death (Burdick et al., 2011). One out of 11 Holstein-Friesian calves died after an i.v. LPS (O111:B4) challenge at 0.5 µg/kg of BW (Plessers et al., 2015). This shows the potential of severe responses of calves to LPS. Furthermore, responses to LPS might differ between calf breeds (Ballou, 2012) and between serotypes of LPS used (Migale et al., 2015). The appropriateness of this challenge depends highly on the objective of a study, and researchers should take potentially severe responses to LPS into account when designing their experiments.

Although this study was not specifically designed to determine the contribution of characteristics of calves in early life to variation in responses to LPS, the data do allow this evaluation. A few measurements performed in early life differed between the heavy and mild responders to LPS. Interestingly, mild responders had a higher presence of fecal pathogens and a lower umbilical score (i.e., more signs of umbilical hernia or inflammation). This might indicate that exposure to pathogens or pathogen-associated molecules (PAMP) in early life attenuate the response to LPS in later life. Comparable observations were done before where environmental exposure to endotoxins was associated with a lower prevalence of asthma in children (Stein et al., 2016) and prior intranasal LPS exposure reduced lung inflammation in ovalbumin-induced asthma in mice (Ding et al., 2018). Further studies are required to assess such relations in calves. Mild responders also had a higher score in the human approach test, indicating more of these calves stopped drinking their milk replacer when a human approached and were thus more reactive. Immune responses of cattle have been associated with behavioral characteristics in previous research. For instance, serum IgG responses to vaccination (Oliphint et al., 2006) and rectal temperature and sickness behavior responses to LPS challenge (Burdick et al., 2011) were lower in temperamental bulls compared with calm bulls.

Even within the group of mild-responding calves, large interindividual variation in clinical responses was observed, with a coefficient of variation of 32% for the maximum increase in rectal temperature and a coefficient of variation of 48% for the maximum increase in respiratory frequency. Large interindividual variation in calves (Michaels and Blanks, 1988) and dairy cows (Jacobsen et al., 2004, 2005) in their clinical response to LPS has been reported before. Mechanisms underlying this large interindividual variation in responses to LPS are largely unknown and we attempted to relate the responses of calves to LPS with other characteristics measured in early life. The maximum increase in rectal temperature was negatively related to the blood Hb concentration measured 4 and 8 wk before the LPS challenge was performed. Hypoferremia has been observed after LPS challenges in dairy cows (Jacobsen et al., 2005). This reduction in blood iron concentration appears to have a functional role in the response to bacterial infection. Proinflammatory cytokines (such as TNF-α, IL-1β, and IL-6) are released in response to an (bacterial) immune challenge, which among others triggers the production of acute phase proteins, with haptoglobin being the main acute phase protein in cattle (Eckersall and Bell, 2010; Ceciliani et al., 2012). Haptoglobin can bind to Hb (Ceciliani et al., 2012), preventing the iron from Hb to be used by bacteria, such as *E. coli*, for their growth and thus preventing pathogen (over)growth. In the current study, potentially, calves with a low Hb concentration before LPS administration reached very low serum iron concentrations following LPS administration, contributing to the severity in clinical response to LPS. However, blood Hb did not correlate with the maximum increase in respiratory frequency, which might be expected due to the role of Hb in oxygen transport. Our data suggest the involvement of early life iron status in later life immune function, but this relation requires further validation. Other early-life health measurements were related to clinical response to LPS as well. The color of the feces related negatively with the maximum increase in respiratory frequency, potentially indicating a connection between the intestine and respiratory response to LPS.

Overall, these data indicate that the responses to a low-dose intravenous LPS challenge (serotype O55:B5; 0.05 μg/kg of BW) vary substantially between calves. Some early-life measurements, mainly hematological and health parameters, were related to the clinical response of calves to LPS. The underlying mechanisms determining individual responses to LPS require further study, but are likely multifactorial. Mortality in this study was high (12.5%) after a low-dose LPS challenge in clinically healthy calves. We, therefore, emphasize that LPS challenges should be used with utmost caution in calf studies.
